# KIAA1429 regulates alternative splicing events of cancer-related genes in hepatocellular carcinoma

**DOI:** 10.3389/fonc.2022.1060574

**Published:** 2022-11-25

**Authors:** Zhao-chen Liu, Lu-Hao Li, Ding-Yang Li, Zhi-Qiang Gao, Dong Chen, Bin Song, Bing-Hua Jiang, Xiao-wei Dang

**Affiliations:** ^1^ Department of Hepatobiliary and Pancreatic Surgery, the First Affiliated Hospital of Zhengzhou University, Zhengzhou, China; ^2^ Center for Genome Analysis, Wuhan Ruixing Biotechnology Co. Ltd, Zhengzhou, China; ^3^ Academy of Medical Sciences, Zhengzhou University, Zhengzhou, China

**Keywords:** alternative splicing, KIAA1429, HCC, RIP, BPTF

## Abstract

Hepatocellular carcinoma (HCC) remains one of the most fatal malignancies with high morbidity and mortality rates in the world, whose molecular pathogenesis is incompletely understood. As an RNA-binding protein participating in the processing and modification of RNA, KIAA1429 has been proved to be implicated in the pathogenesis of multiple cancers. However, how KIAA1429 functions in alternative splicing is not fully reported. In the current study, multi-omics sequencing data were used to analyze and decipher the molecular functions and the underlying mechanisms of KIAA1429 in HCC samples. RNA sequencing data (RNA-seq) analysis demonstrated that in HCCLM3 cells, alternative splicing (AS) profiles were mediated by KIAA1429. Regulated AS genes (RASGs) by KIAA1429 were enriched in cell cycle and apoptosis-associated pathways. Furthermore, by integrating the RNA immunoprecipitation and sequencing data (RIP-seq) of KIAA1429, we found that KIAA1429-bound transcripts were highly overlapping with RASGs, indicating that KIAA1429 could globally regulate the alternative splicing perhaps by binding to their transcripts in HCCLM3 cells. The overlapping RASGs were also clustered in cell cycle and apoptosis-associated pathways. In particular, we validated the regulated AS events of three genes using clinical specimens from HCC patients, including the exon 6 of BPTF gene and a marker gene of HCC. In summary, our results shed light on the regulatory functions of KIAA1429 in the splicing process of pre-mRNA and provide theoretical basis for the targeted therapy of HCC.

## Introduction

Liver cancer ranks sixth among the most frequently-diagnosed cancers and fourth in terms of death ranks, with 75%-85% of cases being caused by hepatocellular carcinoma (HCC) (1). The majority of patients are diagnosed in later stages with poor prognosis. Apart from Hepatitis C virus and Hepatitis B virus infection, HCC progression was associated with several important factors, including inflammation, various molecular events, and different cellular signaling pathways (2). Although there are a variety of therapies, targeted treatment and immunotherapeutic drugs for HCC, drug resistance, tumor recurrence and metastasis still greatly limit the efficacy (3, 4). Therefore, deciphering the key events in cancer progression and thoroughly understanding the mechanisms of liver carcinogenesis will promote the discovery of new targeted drugs.

In pre-messenger RNA (pre-mRNA) splicing, which occurs in post-transcription, introns are spliced to create mature mRNA molecules. Simultaneously, different exons are combined to produce various transcripts. Alternative splicing (AS) defects that are regulated by mutation or in splicing regulatory factors and pre-mRNA sequences, were related to numerous pathologies (5). Dysregulation of AS is a hallmark of human tumors. Moreover, in tumors, these splicing variants specific to cancer are often up-regulated, contributing to cancer development and cancer cell survival, and predicting overall survival time in cancer patients (6, 7). Aberrant alterations of RNA splicing events and splicing factors have been involved in multiple cancers. In cervical neoplasia, the aberrant activity of serine/arginine-rich (SR) proteins and heterogeneous nuclear ribonucleoproteins (hnRNPs) was found to initiate the generation of cancer-causing proteins through processing pre-mRNA transcripts, which were generated from human papillomaviruses genomes or human genes (8). In gastric cancer, overexpressing PTBP3, which was implicated in alternative splicing, may cause inhibition of the differentiation and malignant proliferation of these cells through disrupting the feedback regulation among Hes1, Id1, and PTBP3 (9). Differential AS events (ASEs), which are prevalent in HCC, are largely influenced by the binding relations, expression variations, and even mutations of RNA binding proteins (RBPs) (10). Nuclear-enriched RBP-PTBP3, improves HCC cell metastasis and growth by balancing the splicing variants (NEAT1_1, NEAT1_2 and miR-612) (11). MTR4 drives the metabolic activity of cancer by ensuring that the differential splicing of pre-mRNAs of key glycolytic genes such as GLUT1 and PKM2 is correct (12). Furthermore, prognostic AS signatures have been constructed to predict HCC prognosis and have showed excellent performance in predicting HCC prognosis. Similarly, prognostic AS events were reported to be clustered in metabolism-associated pathways (13, 14).

Among the AS regulators, KIAA1429 is known as an RBP and an important methyltransferase participating in mRNA processing and splicing and m^6^A modification (15). In the cells of mammals, N6-methyladenosine (m^6^A) modification is reversibly regulated by m^6^A writers, erasers, and readers (WERs). Notably, KIAA1429 knockdown is reported to cause m^6^A peak scores to decrease by a median ∼4-fold, which is more conspicuous than that achieved when METTL3 or METTL14 was knocked down, demonstrating that KIAA1429 was essential in the methyltransferase complex (16). Strikingly, accumulating evidence proves that RNA m^6^A modification affects AS events. The m^6^A reader YTHDC1 mediates mRNA splicing by recruiting or blocking the pre-mRNA splicing factors, including SRSF3 and SRSF10 (17), so that those splicing factors can gain or lose access to the binding areas of targeted mRNAs. These discoveries proved the importance of RNA m^6^A modification in regulating AS.

KIAA1429 is involved in the pathogenesis of multiple cancers and its expression is related to the prognostic effect in patients (15, 18, 19). Scientists began to study the functions of KIAA1429 in HCC in recent years. KIAA1429 promoted the invasion and migration of HCC through the inhibition of ID2 *via* the upregulation of m^6^A modification of ID2 mRNA (20). And KIAA1429 was closely associated with the prognostic effect of HCC. A prognostic model which included m^6^A genes (ZC3H13 YTHDF1, YTHDF2, METTL3 and KIAA1429) was developed (21). KIAA1429 facilitates the development of liver cancer through regulating the expression of GATA3 by m^6^A methylation modification (22).

Up until now, the functions of KIAA1429 in AS, tumor genesis and associated mechanisms have not been fully studied. Since methylation is inseparable from alternative splicing and KIAA1429 can bind to RNA and cause m^6^A methylation, we predict that the binding of KIAA1429 to RNAs may regulate the alternative splicing of bound RNAs, thus playing an important role in HCC. Therefore, we downloaded GSE134776 data, which is the transcriptome data obtained from silencing KIAA1429 in HCC cell line HCCLM3. Differentially expressed genes (DEGs) and AS analysis were performed to obtain KIAA1429-regulated AS profile. Then we downloaded GSE134978 data, which is RIP-seq data of KIAA1429 in HCC cell line HCCLM3 with 2 biological duplications. Analysis of KIAA1429-bound RNA was performed. The two sets of data were overlapped to obtain genes that can combine with and be regulated by KIAA1429 for the occurrence of AS events. We extensively studied the function and mechanism of KIAA1429 in liver cancer by deciphering its important roles in AS regulation. This study extended our understanding of KIAA1429 and provided novel insights into the new treatments for HCC in the future.

## Material and methods

### Access to and processing of public data

We used KIAA1429-regulated transcriptome sequencing data (RNA-seq) and its associated RNA sequencing data by the method of RNA sequencing (RIP-seq) and immunoprecipitation (22). The accession numbers of Gene Expression Omnibus (GEO) database were GSE134978 (RIP-seq) and GSE134776 (RNA-seq), respectively. Public sequencing data were obtained from the Sequence Read Archive (SRA). SRA Run data files were transformed into fastq format with NCBI SRA Tool fastq-dump. Low-quality bases were discarded using a FASTX-Toolkit (v.0.0.13). Then the clean reads were analyzed with FastQC.

### Reads alignment and differentially expressed gene analysis

We aligned quality-filtered reads to the human genome (GRCH38) with TopHat2 with at most 4 mismatches (23). Then we used uniquely mapped reads to count the read number and reads per kilobase of exon per million fragments mapped (RPKM) of each gene. Next, we used RPKM to calculate the expression levels of genes. The R package edge R (24), whose function is to identify which genes are differentially expressed, was applied to identify DEGs from RNA-seq data. The false discovery rate (FDR ≤ 0.05) and fold change (FC≥2 or ≤0.5) were applied to determine whether a gene was significantly differential between siKIAA1429 and control.

### Alternative splicing analysis

ABLas pipeline was applied to define and quantify alternative splicing events (ASEs) and regulated alternative splicing events (RASEs) by siKIAA1429 as described previously (25, 26). Briefly, on the basis of the splice junction reads, we detected ten types of alternative splicing events, including exon skipping (ES), alternative 3’splice site (A3SS), alternative 5’ splice site (A5SS), A3SS&ES, A5SS&ES, mutually exclusive exons (MXE), intron retention (IR), cassette exon, mutually exclusive 5’UTRs (5pMXE), and mutually exclusive 3’UTRs (3pMXE).

For sample pair comparison, Fisher’s exact test was applied to determine statistical significance. The p-value <=0.05 and RASE ratio >=0.2 were set as the threshold to detect RASEs. For repetition comparison, Student’s *t*-test was conducted to evaluate the significance of AS ratio alteration. Those events with p-value <= 0.05 were regarded as RASEs.

### RIP-seq data analysis

For KIAA1429 RIP-seq data, the reads-mapping and quality-filtering methods were the same as RNA-seq data. After the uniquely mapped reads were aligned onto the genome sequences, we used random IP method to detect the binding sites (peaks) of KIAA1429 on transcripts, which had been fully described in a previous study (26). After filtering the binding peaks of KIAA1429 with *p*-value < 0.05 as criterion, we extracted the sequences of peaks and detected the enriched motifs using HOMER software (27).

### Functional enrichment analysis

We used Gene Ontology (GO) terms, KEGG pathways and Reactome pathways to identify functional categories of DEGs *via* KOBAS 2.0 server (28). The statistical significance was adjusted by Benjamini-Hochberg FDR.

### Validating alternative splicing events by qPCR

We further validated the alternative splicing events of AS genes (URI1, MTMR14, BPTF) regulated by KIAA1429 in clinical samples using RT-qPCR. We extracted 13 pairs liver cancer tissues and adjacent normal tissues of HCC patients from the First Affiliated Hospital of Zhengzhou University and examined the AS levels of three genes selected. The studies involving human participants were reviewed and approved by the ethics committee of the First Affiliated Hospital of Zhengzhou University. All methods were employed according to the regulations and guidelines. We strictly conformed to the biosecurity law and followed institutional safety procedures in China.All the specimens were processed instantly after being collected, and then stored at − 80°C for RNA extraction. TRIzol reagent (Invitrogen) was used to extract toal RNAs. Then PrimeScript RT reagent Kit (Takara) was used to convert 10 μg RNA into complementary DNA (cDNA). RT-qPCR was conducted according to the published method (29, 30).

### Other statistical analysis

After the reads of each gene were normalized by TPM (Tags per Million), in-house script (sogen) was used to visualize genomic annotations and next-generation sequence data. Based on Euclidean distance, the pheatmap package (https://cran.rproject.org/web/packages/pheatmap/index.html) in R was used to perform the clustering the two groups were compared using Student’s *t*-test.

## Result

### KIAA1429 is highly expressed in HCC and negatively associated with the prognosis of patients

**Figure 1:**
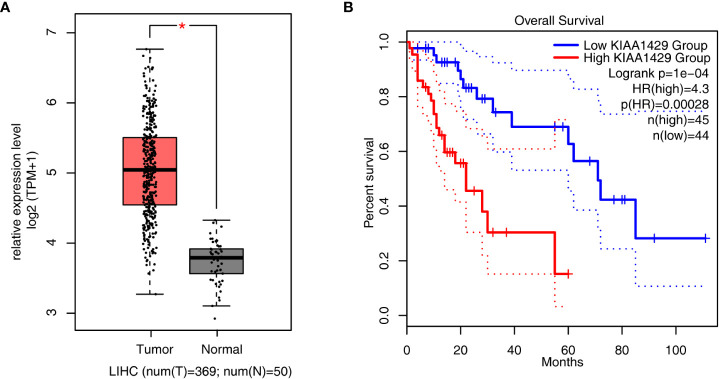
Analysis of KIAA1429 expression levels and prognosis in liver hepatocellular carcinoma samples from TCGA database. **(A)** Boxplot showing the expression level of KIAA1429 in 419 liver hepatocellular carcinoma samples from TCGA database, including 50 normal samples and 369 tumor samples. Error bars represent mean ± SEM. *p < 0.05. **(B)** Overall survival (OS) rate of HCC patients with high expression of KIAA1429 (top 25% of the expression range) versus low expression of KIAA1429 (bottom 25% of the expression range).

To have an overview of the expression level and prognosis effect of KIAA1429 in liver cancer, we downloaded RNA-seq expression data of 419 samples in TCGA (The Cancer Genome Atlas) database, including 369 tumor samples and 50 normal samples. Then the expression level of KIAA1429 was analyzed. The result showed that KIAA1429 was higher expressed in 369 tumor samples compared with in 50 normal tissue samples with significance (**Figure 1A**). We further identified the relationship between the survival rate in HCC patients and the expression level of KIAA1429. The overall survival (OS) rate of patients in low KIAA1429 group was higher than that in high KIAA1429 group (**Figure 1B**), demonstrating that high expression of KIAA1429 may lead to a poor prognosis inHCC. To explore the expression level of KIAA1429 in multiple cancers, we used GEPIA2 online tool (31) and found that the expression levels of KIAA1429 were higher in multiple tumors (**Figure S1A**). Survival rate analysis showed that higher expression of KIAA1429 was correlated with worse prognosis in multiple cancer types (**Figure S1B**). These results indicate that KIAA1429 performs important functions in the progression of HCC.

### RNA Immunoprecipitation sequencing analysis showed the RNA binding features of KIAA1429 in HCCLM3 Cells

**Figure 2:**
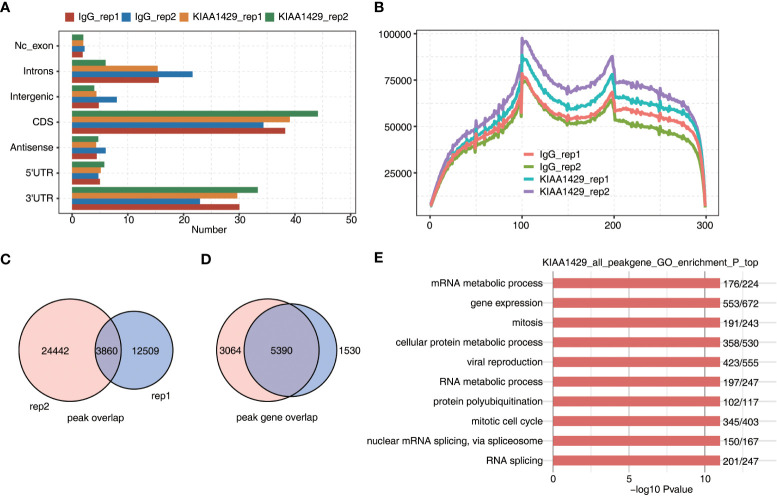
RIP-seq data demonstrated the RNA interactome of KIAA1429 in HCCLM3 cells. **(A)** The distribution of genomic region of KIAA1429-bound peaks. were shown by bar plot. **(B)** The peak reads distribution on transcripts was shown by line plot. Three regions (5’UTR, CDS, and 3’TUR) in each gene were divided into 100 bins. The KIAA1429 peak reads of each bin were counted. The reads density of KIAA1429 peaks in each gene was presented. **(C)** The overlapping peaks in two replicate RIP-seq samples were shown by venn plot. **(D)** The overlapping KIAA1429-bound genes in two replicate RIP-seq samples were shown by venn plot. **(E)** The top 10 enriched GO biological processes of genes bound by KIAA1429 were shown by bar plot.

To further investigate how KIAA1429 functions in HCC, we downloaded RIP-seq data of KIAA1429 in HCC cell line HCCLM3 (GSE134978), and analyzed the global RNA binding features of KIAA1429. After the quality-filtered reads were aligned onto human genome, we found that KIAA1429 showed higher percentage in coding DNA sequence (CDS) and 3’ untranslated regions (UTR) regions compared with IgG control, and reads aligned in 3’UTR and CDS regions occupied over 70% of total aligned reads (**Figure 2A**). We then divided CDS, 5’UTR, and 3’UTR into 100 portions for each region and analyzed the reads distribution in these regions. The results showed that there was one peak around start codon and one around stop codon. Meanwhile, CDS and 3’UTR regions also showed higher levels in KIAA1429 IP samples compared with in IgG (**Figure 2B**), consistent with the results in **Figure 2A**. We then systematically identified the binding sites (peaks) of KIAA1429 using previously described method (26). A total of 28896 and 16544 peaks were detected from replicate 1 and 2, respectively; and 3895 peaks were shared by the two replicates (**Figure 2C**). Following analysis of overlapping genes bound by KIAA1429 in two biological replicates revealed that up to 5524 genes were shared by the two replicates (**Figure 2D**). After functional enrichment analysis was conducted, these overlapping genes were found to be clustered in GO BP pathways including RNA metabolism, mRNA metabolism, cell protein metabolism, mitosis, mitotic cell cycle, viral reproduction, nuclear mRNA splicing *via* spliceosome, RNA splicing, and DNA repair (**Figure 2E**), indicating KIAA1429 could bind to numerous RNAs with essential functions in HCC HCCLM3 cells. Similar analysis using KEGG pathway database also revealed that bound genes were highly enriched in RNA splicing and cell cycle-associated pathways (**Figure S2A**). Furthermore, we analyzed the enriched motif sequences among the KIAA1429-bound peaks using HOMER software (27), the results of which showed that the specific binding motif of KIAA1429 was UCGAUG in two biological replicates (**Figure S2B**). In summary, the RIP-seq confirmed that KIAA1429 had the ability to bind multiple target RNAs and had potential effects on the targeted RNAs in HCC cells.

### KIAA1429-mediated alternative splicing events in HCCLM3 cells

**Figure 3:**
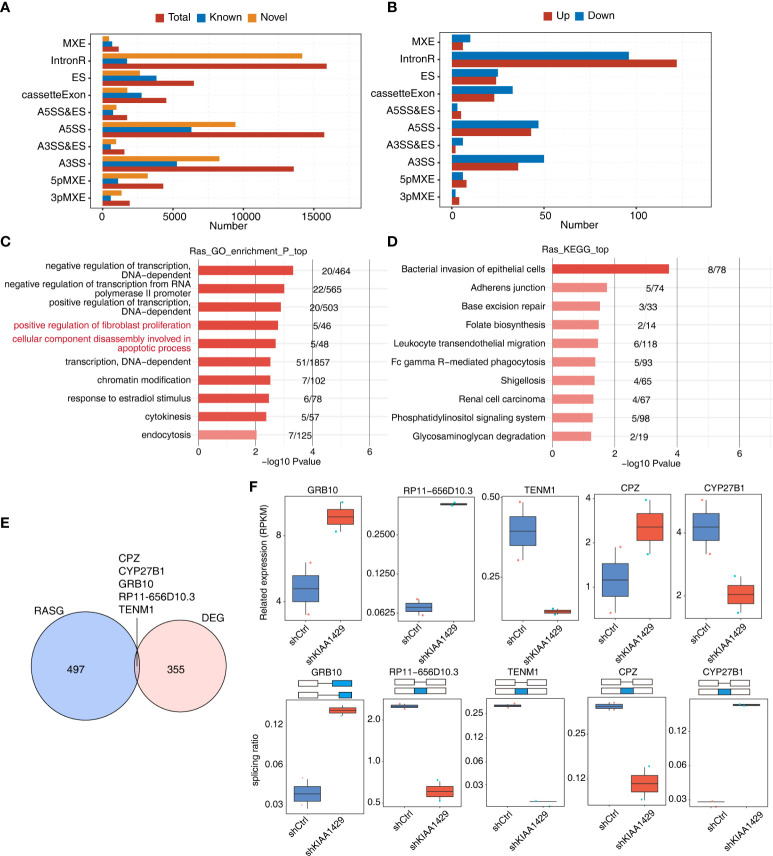
KIAA1429-regulated alternative splicing events in HCCLM3 cells. **(A)** Classification of all the AS events detected. X-axis: Percentage. **(B)** Classification of all the AS events (RAS) regulated by KIAA1429. X-axis: AS number. up: alternative splicing pattern was up-regulated compared with model splicing pattern. down: alternative splicing pattern was down-regulated compared with model splicing pattern. **(C)** The top 10 enriched GO biological processes of alternative splicing genes regulated by KIAA1429. **(D)** The top 10 enriched KEGG pathways of alternative splicing genes regulated by KIAA1429. **(E)** Venn diagram displaying the overlap of differential expression genes (DEGs) and alternative splicing genes (RASGs) regulated by KIAA1429. **(F)** Box plots shows expression levels (left) and PSI profiles of two examples of overlapping genes from **(E)**.

To further explore the outcoming influence of KIAA1429 on its bound transcripts in HCCLM3 cells, we downloaded the global transcriptional sequencing (RNA-seq) data (GSE134776) of KIAA1429-silenced and control HCCLM3 cells with 2 biological duplicates, which were generated using the same cell batch of HCCLM3 RIP-seq data. After aligning RNA-seq data onto genome and calculating gene expression levels and splicing junction reads, we performed DEGs and alternative slicing AS analyses to obtain DEGs and AS events regulated by KIAA1429. Since the previous study of the RNA-seq data focused on DEGs (22), in this study we mainly investigated the RASEs of KIAA1429. Using ABLas program (26), we analyzed the alternative splicing events of the RNA-seq dataset and investigated the ratio changes in AS occurrence. Besides the known AS events previously annotated in the annotation file, we discovered plenty of novel alternative splicing events and classified them into ten canonical AS types. Dominant alternative splicing types include IR, ES, A5SS and A3SS (**Figure 3A**). Then we extracted RASEs with significant differences (p-value < 0.05) between siKIAA1429 samples and control samples, and detected hundreds of RASEs after silencing KIAA1429 (**Figure 3B**). The changed ratios of these RASEs showed consistent patterns in the two biological replicates (**Figure S3A**). Functional enrichment analysis of genes from RASEs revealed that these genes mediated by KIAA1429 at AS level were highly enriched in the positive regulation of fibroblast proliferation and the decomposition of cell components during apoptosis (**Figure 3C**), which were closely related to tumor progression. Transcription-related pathways were also enriched (**Figure 3C**). KEGG and Reactome pathway analyses demonstrated several pathways associated with tumor progression (**Figure 3D**, **Figure S3B**). Our DEG and RASG overlapping analyses showed that only 5 genes are both DEGs and RASGs, including CPZ, CYP27B1, GRB10, RP11-656D10.3, and TENM1 (**Figure 3E**). We then illustrated the expression levels and splicing ratio changes of these 5 genes and found consistent changes between siKIAA1429 and control (**Figure 3F**). In summary, these results indicate that KIAA1429 could regulate transcriptome profile through transcriptional and post-transcriptional regulation manners.

### KIAA1429 selectively binds to mRNAs to regulate alternative splicing of cancer-associated genes

**Figure 4:**
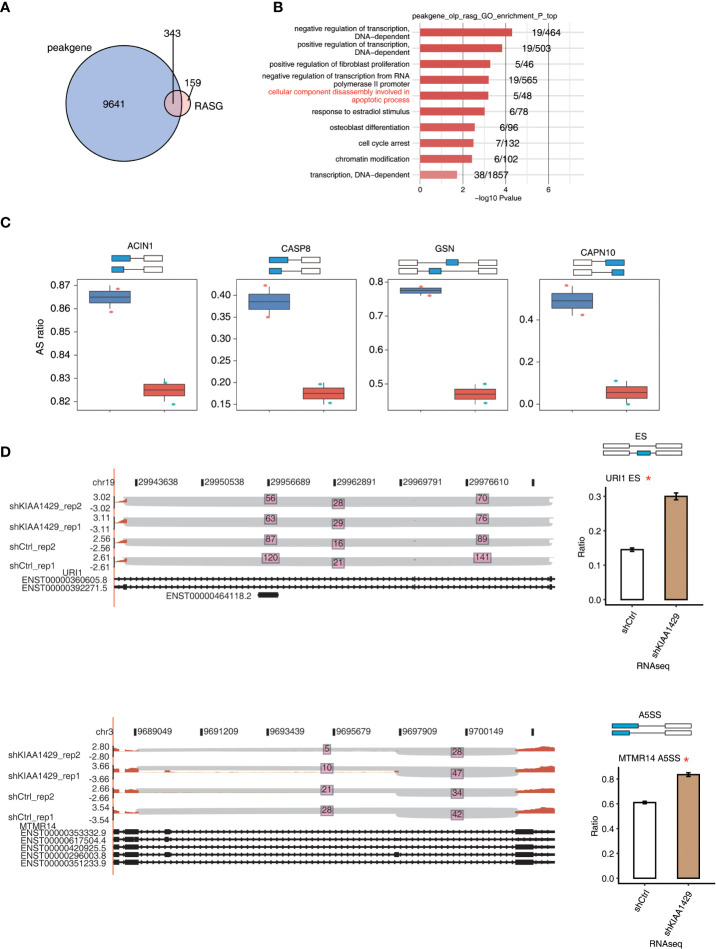
KIAA1429 selectively binds to mRNA to regulate alternative splicing of cancer related genes. **(A)** Venn diagram showed the overlap of KIAA1429-bound genes and KIAA1429-regulated alternatively splicing genes. **(B)** The top 10 enriched GO biological processes of the genes of overlap of KIAA1429-bound peaks and -regulated alternatively splicing events. **(C)** Bar plots shows PSI profile of the four overlapped KIAA1429-regulated alternative splicing events by RNA binding in HCCLM3 Cells. **(D)** IGV-sashimi plot showed the KIAA1429-regulated alternative splicing events across mRNA of URI1 and MTMR14. Reads distribution of each alternative splicing event was plotted in the left panel with the transcripts of each gene shown below. The schematic diagrams depict the structures of ASEs at the top of the right panel. RNA-seq quantification of ASEs is shown at the bottom of the right panel. Error bars represent mean ± SEM. *p < 0.05.

To further explore if there is an association between KIAA1429-bound transcripts and KIAA1429-regulated ASEs, we conducted an interaction analysis. Although there were only four RASEs that had KIAA1429-bound peaks around their genomic locations (**Figure S4A**), we found that about 67% (340/502) of alternative splicing genes regulated by KIAA1429 overlapped with genes bound by KIAA1429 (p-value ≤ 0.05, Hypergeometric test, **Figure 4A**), suggesting that KIAA1429 might regulate a large number of alternative splicing events through directly binding to RNA targets. Functional enrichment analysis of these overlapping genes demonstrated that they were highly enriched in several functional pathways, including decomposition of cellular components during apoptosis, cell cycle block, etc., which were closely related to liver cancer (**Figure 4B**). Functional enrichment analysis of KEGG and Reactome databases also revealed several enriched pathways (**Figure S4B, C**). We then selected and presented the splicing ratio changes of several RASGs that were from apoptotic process and were also bound by KIAA1429, including ACIN1, CASP8, GSN, and CAPN10 (**Figure 4C**). The splicing reads number and ratio changes of URI1 (ES event), MTMR14 (A5SS event), and BPTF (ES event) were also presented to show the significant differences between siKIAA1429 and control (**Figure 4D** and **Figure S4D**).

### Validation of alternative splicing events regulated by KIAA1429 in liver cancer using clinical specimens

**Figure 5:**
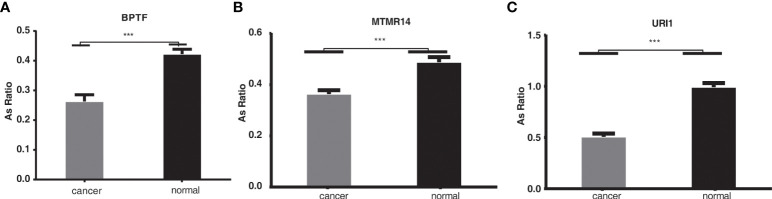
The validation of the alternative splicing events in clinical specimens. ***p < 0.001. **(A–C)**: The RT-qPCR results revealed that AS ratios of these three genes significantly decreased in cancer samples (p < 0.001).

We then validated the identified RASEs using clinical specimens from liver cancer patients by RT-qPCR experiment. We randomly selected three alternative splicing events regulated by KIAA1429, including URI1 (ES event), MTMR14 (A5SS event), and BPTF (ES event), which were shown in **Figure 4** and **Figure S4**. Specific primers for RASEs were designed according to the exact splicing junction sequences (32). The AS ratios of these three AS events all increased in siKIAA1429 samples, indicating that they should increase in normal clinical specimens from which the expression level of KIAA1429 decreased compared with that of HCC samples (**Figure 1A**). The RT-qPCR results revealed that AS ratios of these three genes significantly decreased in cancer samples (p < 0.001, **Figures 5A-C**), conforming to the RNA-seq results in KIAA1429-silenced HCCLM3 cells.

## Discussion

As a multifunctional RNA binding protein and an important RNA methyltransferase, KIAA1429 is implicated in mRNA splicing and processing and m^6^A modification (15), and the altered KIAA1429 function enhances the proliferation, migration and invasion abilities of HepG2 cells through the inhibition of ID2 *via* the upregulation of m^6^A modification of ID2 mRNA (20). Additionally, increasing studies proved that KIAA1429 is related to the progression of multiple cancers, such as gastric cancer (19), osteosarcoma (18), and breast cancer (15). Thus, high-throughput methods have been adopted to identify targets of KIAA1429. KIAA1429 facilitates liver cancer progression by regulating the expression of GATA3 through m^6^A methylation modification (22). In this study, RNA immunoprecipitation sequence (RIP-seq) and RNA-seq data were applied to draw a comparison between the expression profiles of control and KIAA1429 stable knockdown HCC cells, the analyzing results of which, demonstrated that KIAA1429 can affect the alternative splicing patterns of genes implicated in various pathways by binding to numerous RNAs. Our results highlight the important regulatory roles of KIAA1429 in AS pattern. Thus, KIAA1429 might affect the development of HCC and could serve as a therapeutic target in the future.

The RIP-seq method was an approach to systematically identify the transcripts to which an RBP binds (33). In this study, RIP-seq analysis demonstrated that the RNA binding motif of KIAA1429 is UCGAUG. AS genes bound by KIAA1429 are enriched in functional pathways that are closely related to tumors, including mRNA metabolism, RNA metabolism, cell protein metabolism, mitosis, mitotic cell cycle, viral reproduction, nuclear mRNA splicing *via* spliceosome, RNA splicing, and DNA repair. This discovery greatly broadens our understanding of the functions of KIAA1429 in various biological processes. These results demonstrated that KIAA1429 functions in liver cancer mainly by regulating AS events. Previous studies have proved that KIAA1429 plays a vital part in the progression of liver cancer, but the functions of KIAA1429 in AS and tumorigenesis and associated mechanisms are still unclear. Our DEG and AS analysis shows that only 5 genes were differentially expressed and variably spliced simultaneously, indicating that KIAA1429 regulates distinct gene sets through transcriptional and post-transcriptional regulation. We hypothesized that KIAA1429 may, through its methylation, alter the variable splicing of key tumor genes and generate tumor-promoting spliceosomes, thus promoting tumor progression. In the present study, AS events regulated by KIAA1429 occurring in these genes were greatly enriched in the positive regulation of fibroblast proliferation and the decomposition of cell components during apoptosis, which were closely related to tumor progression. Meanwhile, a recent study demonstrated that key RNA methyltransferase METTL3 could promote tumorigenesis by enhancing translation of epigenetic factors in the absence of m^6^A (34). We propose that KIAA1429 may also regulate AS patterns independent of m^6^A modification in primary-RNAs.

Furthermore, recent study demonstrated that neoantigens for therapies based on immune in cancer can arise from dysregulated splicing (35). Since immune checkpoint inhibitors (e.g., ipilimumab and nivolumab) and chimeric antigen receptors (CAR-T) have succeeded in increasing patient survival, cancer therapies had been revolutionized by immunotherapy-based treatments (36, 37). Using RBP-focused CRISPRCas9 screening, researchers uncovered an essential role of YTHDF2 in breast cancer driven by MYC, highlighting the important functions of RBPs serving as effective therapeutic targets (38).Their studies revealed that RBP-RNA interactions promoted the progression of diseases and facilitated the growth and survival of tumor cells instead of somatic tissues, and that targeting RBPs is expected to be safe and effective and precise treatment modalities in particular cancer subtypes (38).For this reason, more studies are urgently needed to examine the immunogenicity of underlying neoantigens derived from AS events regulated by KIAA1429 for immunotherapies in liver cancer.

In addition, due to dysregulated splicing in cancer, oncogenes have undergone isoform switching as a mechanism through which cancer cells developed drug resistance to cancer treatments. For example, melanoma patients acquire resistance to RAF inhibitors through generating spliced isoforms of BRAF V600E in the absence of RAS-binding domain (39). Additionally, chronic myeloid leukemia patients with BCR-ABL chromosomal translocation displayed alternative splicing variants of BCR-ABL when treated with imatinib, the tyrosine kinase inhibitor (40). AS events regulated by KIAA1429 occurring in these genes were clustered in the positive regulation of the decomposition of cell components during apoptosis, suggesting that KIAA1429 may play a vital part in tumor drug resistance through regulating the splicing of related genes, which requires further exploration.

Our study also demonstrated that by directly binding to RNA targets, KIAA1429 might regulate many alternative splicing events. Several pathways emerged from the functional analysis of these genes: decomposition of cellular components during apoptosis, cell cycle block, etc., which are closely related to liver cancer. The 3 randomly selected alternative splicing genes (URI1, MTMR14 and BPTF) were further validated by qPCR in HCC clinical samples and alternative splicing events of these three genes are significantly changed (p < 0.001). Among them, BPTF is a protein-coding gene, about which researchers in neurodegenerative diseases are extremely concerned (such as Alzheimer’s disease) (41). In patients with neurodegenerative diseases, high expression levels of BPTF have been detected. In recent years, the role of BPTF in tumor has caught the attention of a large number of researchers. BPTF is found to be highly expressed in HCC. And high expression of BPTF leads to poor overall survival, which may be related to epithelial-mesenchymal transition (EMT) (42). Previous studies have shown that BPTF knockout also inhibits the growth and metastasis of HCC tumors in xenograft mouse models, and that BPTF may have the potential to be a new target for hepatocellular carcinoma therapy (43). Zhang et al. recently investigated the vital role of m^6^A modification and the METTL14/BPTF axis in the epigenetic and metabolic remodeling of metastasis of renal cell carcinoma, highlighting the BPTF inhibitor-AU1 as a key therapeutic candidate (44). Our preliminary study found that KIAA1429 inhibited the generation of BPTF variant splice subtype BPTF-A, but promoted the generation of BPTF-B, so we speculated that BPTF-B could have greater influence on HCC metastasis than BPTF-A, which needs future validation. However, our study is based on the RNA-seq and RIP-seq data from single cell line, which should be validated experimentally for further analysis. Further studied are urgently needed to reveal its specific and detailed mechanism, by which KIAA1429 regulate many alternative splicing events.

In summary, as an RBP, KIAA1429 is aberrantly expressed in HCC, whereas its role as a safe and effective drug target remains largely unexplored. Our study revealed that KIAA1429 regulated alternative splicing events by binding to transcripts which were tightly associated with cell cycle and apoptosis in live cancer. This functional manner of KIAA1429 may be achieved by m^6^A modification of RNAs, or perhaps by regulating m^6^A modification of host RNAs. Our results extended the understanding of the molecular mechanisms of KIAA1429 in HCC cells, demonstrating that KIAA1429 may be a potential molecular target for the development of new therapeutics for liver cancer treatment in the future.

## Data availability statement

The original contributions presented in the study are included in the article/**Supplementary Material**. Further inquiries can be directed to the corresponding author.

## Ethics statement

The studies involving human participants were reviewed and approved by the ethics committee of the First Affiliated Hospital of Zhengzhou University. The patients/participants provided their written informed consent to participate in this study.

## Author contributions

B-HJ and XD designed and coordinated the project. D-YL and Z-QG performed the experimental research and analyzed the data. DC and BS performed bioinformatics analysis. ZL and L-HL wrote the manuscript. All authors commented on or contributed to the final manuscript.

## Funding

This study was supported by Joint project of Henan Province Medical Science and Technology Program for Tackling Key Problems (LHGJ20190035).

## Acknowledgments

The authors acknowledge the infrastructure and staff support provided by Center for Genome Analysis, Wuhan Ruixing Biotechnology Co. Ltd.

## Conflict of interest

Authors DC and BS were employed by Wuhan Ruixing Biotechnology Co. Ltd

The remaining authors declare that the research was conducted in the absence of any commercial or financial relationships that could be construed as a potential conflict of interest.

## Publisher’s note

All claims expressed in this article are solely those of the authors and do not necessarily represent those of their affiliated organizations, or those of the publisher, the editors and the reviewers. Any product that may be evaluated in this article, or claim that may be made by its manufacturer, is not guaranteed or endorsed by the publisher.

## Supplementary material

Supplementary Figure 1 Analysis of the expression levels and prognosis of KIAA1429 in different tumor samples from TCGA database. **(A)** Relative expression (TPM) of KIAA1429 in tumor samples (red) from 16 cancer types versus normal samples (green) *P < 0.05. **(B)** Correlation of KIAA1429 expression with the survival rates in multiple cancer types.

Supplementary Figure 2 RIP-seq data showed the RNA interactome data of KIAA1429 in HCCLM3 cells. **(A)** the top 10 enriched GO biological processes of KIAA1429-bound genes were shown by bar plot. **(B)** Top five motifs of KIAA1429-bound peaks using HOMER software.

Supplementary Figure 3 KIAA1429-mediated alternative splicing events in HCCLM3 cells. **(A)** PSI heatmap of all significantly regulated alternative splicing events (intron retention events, IR RAS) among KIAA1429-KD and control samples. **(B)** The top 10 enriched Reactome pathways of alternative splicing genes regulated by KIAA1429.

Supplementary Figure 4 KIAA1429 selectively binds to mRNA for regulating the alternative splicing of cancer-related genes. **(A)** The overlap of peaks bound by KIAA1429 and alternatively spliced events regulated by KIAA1429 was shown in venn diagram. **(B)** The top 10 enriched KEGG pathways of the overlapping genes of peaks bound by KIAA1429 and alternatively spliced events regulated by KIAA1429. **(C)** The top 10 enriched Reactome pathways of the overlapping genes of peaks bound by KIAA1429 and alternatively spliced events regulated by KIAA1429. **(D)** The alternative splicing events regulated by KIAA1429 across mRNA of BPTF were shown in IGV-sashimi plot. In the left panel was the reads distribution of each alternative splicing event and the transcripts of each gene were shown below. At the top of the right panel were the schematic diagrams depicting the structures of ASEs. At the bottom of the right panel was RNA-seq quantification of ASEs. Error bars represent mean ± SEM. *p < 0.05.

## Abbreviations

AS, alternative splicing, RASGs, Regulated AS genes, RNA-seq, Transcriptome sequencing data, RIP-seq, RNA Immunoprecipitation sequencing, HCC, hepatocellular carcinoma, pre-mRNA, pre-messenger RNA, DEG, differentially expressed gene, RBPs, RNA binding proteins, m6A, N6-methyladenosine, GEO, Gene Expression Omnibus, ES, exon skipping, A3SS, alternative 3’splice site, A5SS alternative 5’ splice site, MXE, mutually exclusive exons, IR, intron retention, 5pMXE, mutually exclusive 5’UTRs, 3pMXE, mutually exclusive 3’UTRs, ASEs, alternative splicing events, LIHC, liver hepatocellular carcinoma.
