# Supplementary figures and images for: KIAA1429 regulates alternative splicing events of cancer-related genes in hepatocellular carcinoma
Source: Front Oncol. 2022 Nov 25;12:1060574. doi: 10.3389/fonc.2022.1060574 (PMC9732450; doi:10.3389/fonc.2022.1060574)

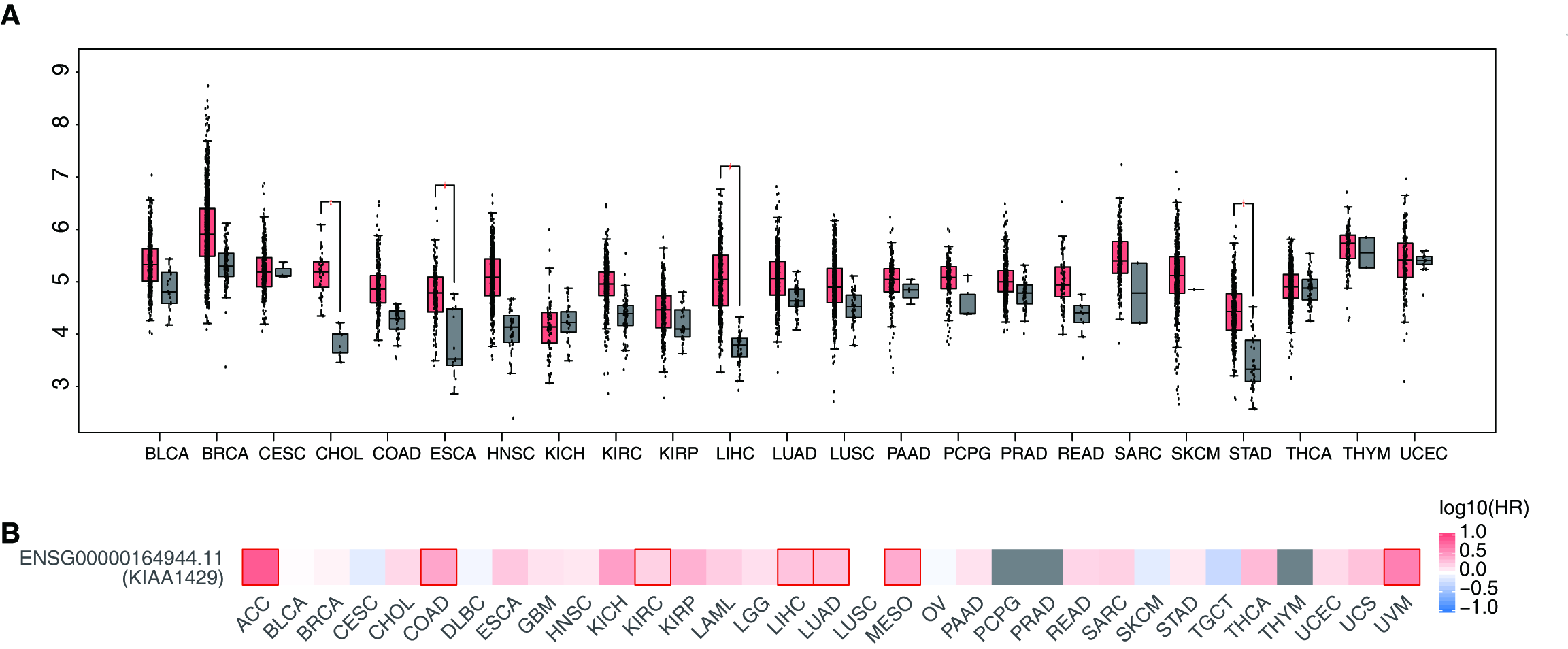

Supplement: Supplementary Figure 1 — Analysis of the expression levels and prognosis of KIAA1429 in different tumor samples from TCGA database. (A) Relative expression (TPM) of KIAA1429 in tumor samples (red) from 16 cancer types versus normal samples (green) *P < 0.05. (B) Correlation of KIAA1429 expression with the survival rates in multiple cancer types. [file Image_1.tif]

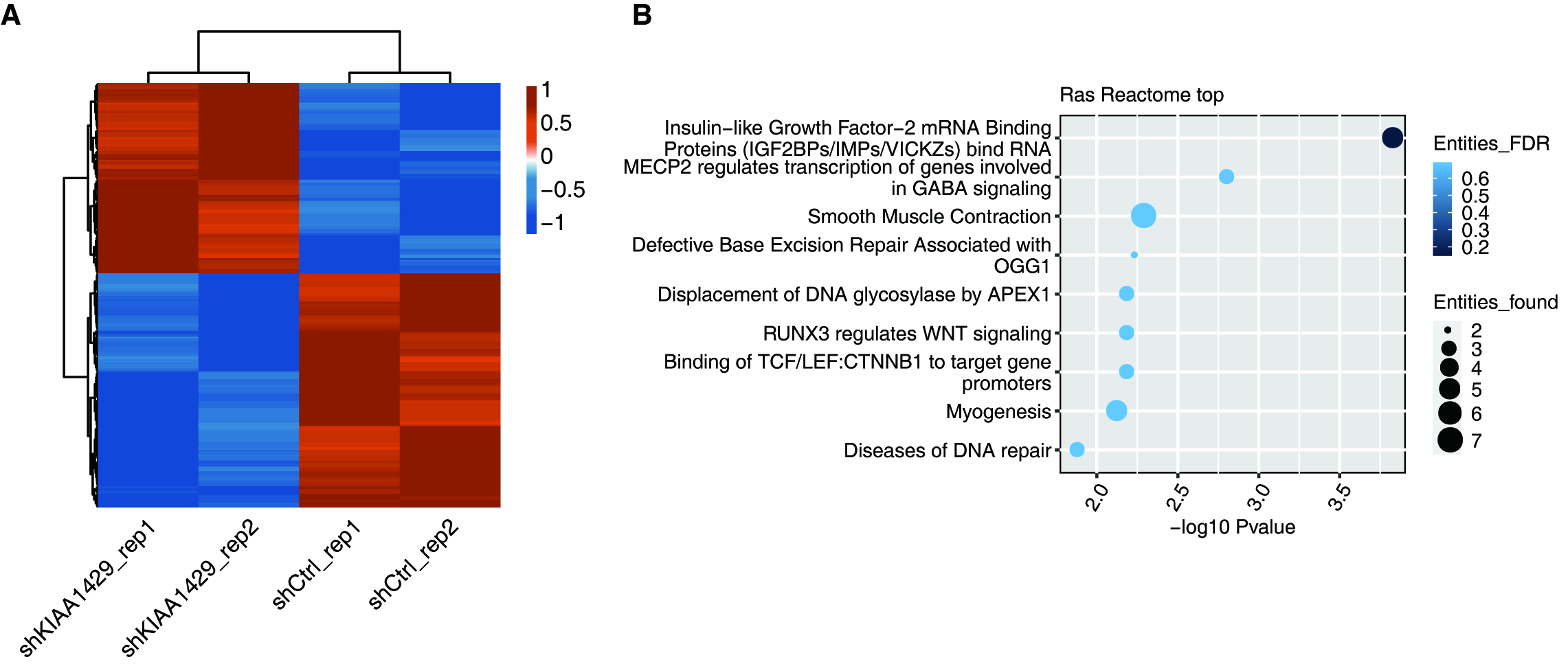

Supplement: Supplementary Figure 2 — RIP-seq data showed the RNA interactome data of KIAA1429 in HCCLM3 cells. (A) the top 10 enriched GO biological processes of KIAA1429-bound genes were shown by bar plot. (B) Top five motifs of KIAA1429-bound peaks using HOMER software. [file Image_2.tif]

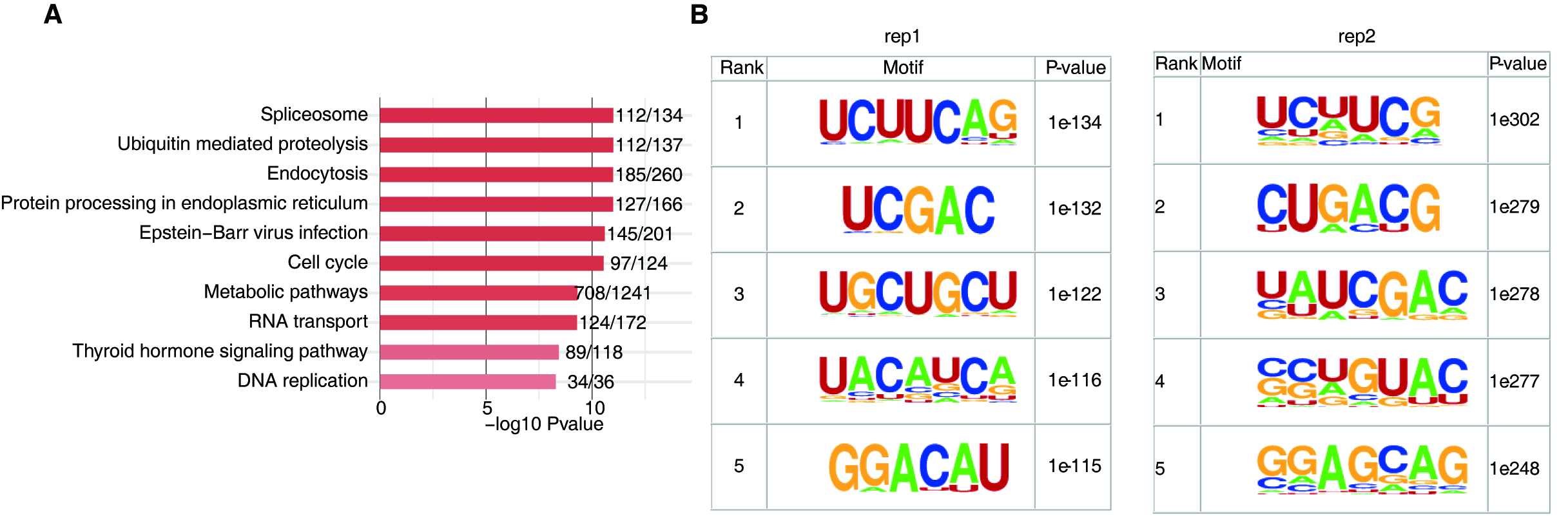

Supplement: Supplementary Figure 3 — KIAA1429-mediated alternative splicing events in HCCLM3 cells. (A) PSI heatmap of all significantly regulated alternative splicing events (intron retention events, IR RAS) among KIAA1429-KD and control samples. (B) The top 10 enriched Reactome pathways of alternative splicing genes regulated by KIAA1429. [file Image_3.tif]

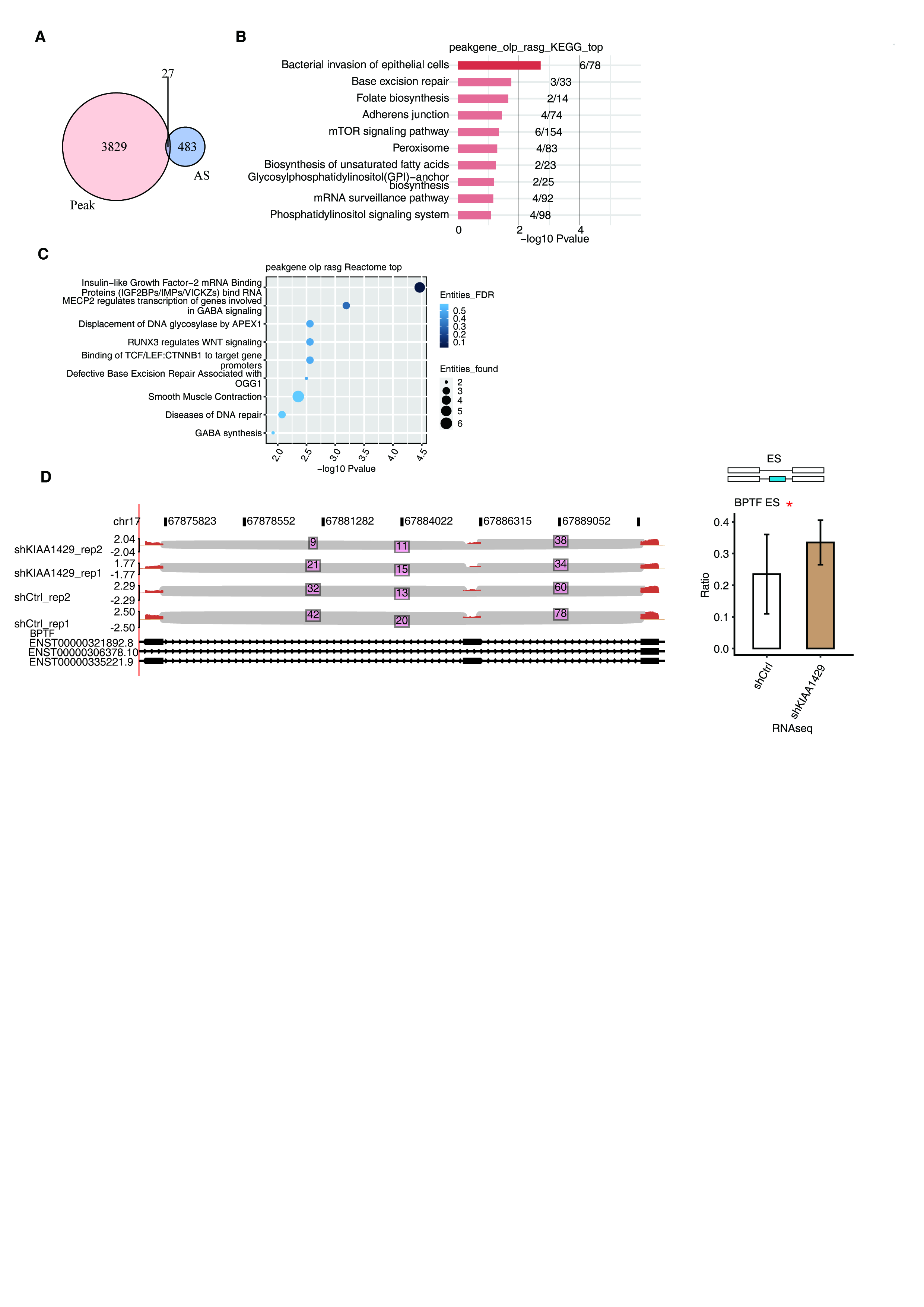

Supplement: Supplementary Figure 4 — KIAA1429 selectively binds to mRNA for regulating the alternative splicing of cancer-related genes. (A) The overlap of peaks bound by KIAA1429 and alternatively spliced events regulated by KIAA1429 was shown in venn diagram. (B) The top 10 enriched KEGG pathways of the overlapping genes of peaks bound by KIAA1429 and alternatively spliced events regulated by KIAA1429. (C) The top 10 enriched Reactome pathways of the overlapping genes of peaks bound by KIAA1429 and alternatively spliced events regulated by KIAA1429. (D) The alternative splicing events regulated by KIAA1429 across mRNA of BPTF were shown in IGV-sashimi plot. In the left panel was the reads distribution of each alternative splicing event and the transcripts of each gene were shown below. At the top of the right panel were the schematic diagrams depicting the structures of ASEs. At the bottom of the right panel was RNA-seq quantification of ASEs. Error bars represent mean ± SEM. *p < 0.05. [file Image_4.tif]
